# Fidgetin knockdown and knockout influences female reproduction distinctly in mice

**DOI:** 10.7555/JBR.36.20220086

**Published:** 2022-07-10

**Authors:** Cong-Rong Li, Ruo-Lei Wang, Shi-Ya Xie, Yan-Ru Li, Lei-Lei Gao, Zhi-Xia Yang, Dong Zhang

**Affiliations:** 1 State Key Lab of Reproductive Medicine, Nanjing Medical University, Nanjing, Jiangsu 211166, China; 2 Animal Core Facility, Nanjing Medical University, Nanjing, Jiangsu 211166, China

**Keywords:** mouse, fidgetin, knockdown, knockout, female reproduction

## Abstract

Microtubule-severing proteins (MTSPs), are a family of proteins which use adenosine triphosphate to sever microtubules. MTSPs have been shown to play an important role in multiple microtubule-involved cellular processes. One member of this family, fidgetin (*FIGN*), is also involved in male fertility; however, no studies have explored its roles in female fertility. In this study, we found mouse fidgetin is rich within oocyte zona pellucida (ZP) and is the only MTSP member to do so. Fidgetin also appears to interact with all three ZP proteins. These findings prompted us to propose that fidgetin might prevent polyspermy. Results from *in vitro* maturation oocytes analysis showed that fidgetin knockdown did cause polyspermy. We then deleted all three fidgetin isoforms with CRISPR/Cas9 technologies; however, female mice remained healthy and with normal fertility. Of all mouse MTSPs, only the mRNA level of fidgetin-like 1 (*FIGNL1*) significantly increased. Therefore, we assert that fidgetin-like 1 compensates fidgetin's roles in fidgetin knockout female mice.

## Introduction

Microtubule-severing proteins (MTSPs) are a family of proteins which use adenosine triphosphate to sever microtubules^[1–2]^. MTSPs have been found to play important roles in multiple microtubule-involved cellular processes, including mitosis, morphogenesis, motility, protein and organelle transport, signaling, and multiple neuronal activities^[3–6]^. In humans, mutations in MTSP, namely spastin, has been closely associated with autosomal dominant spastic paraplegia, which is characterized by progressive weakening of lower limbs induced by the degeneration of corticospinal tracts^[7]^.

While a large number of studies have shown the importance of MTSPs in mitotic cells, less research has focused on analyzing the role of MTSPs in meiotic reproductive cells such as oocytes or spermatocytes. For example, katanin-like 1 (KL1) is expressed in testicular Sertoli cells (SCs) from 15.5 days post-coitum, and KL1 loss of function contributes to male-specific infertility by disrupting SC microtubule dynamics and initiating premature release of spermatids from the seminiferous epithelium^[8]^. Simultaneously, KL2 is also enriched in the testis and has numerous functions, including sperm head shaping, axoneme initiation, and sperm release. However, distinct from KL1, KL2 functional loss (or knockout) almost completely eliminates mature sperm in the epididymis^[9]^. Additionally, KL2 interacts with δ- and ε-tubulin instead of severing microtubules composed of α- and β-tubulin^[9]^.

Oocytes in the ovaries are key cells involved in reproductive processes and undergo meiotic maturation upon luteinizing hormone surge. Fidgetin was so called because mutation in *FIGN* caused a "fidget-like" phenotype characterized by a side-to-side head-shaking and circling behavior due to defective semicircular canals. Fidget mice also have a cell-cycle delay, with small eyes due to insufficient growth of the retinal neural epithelium, and fewer penetrance skeletal abnormalities^[10–11]^. However, little is known about fertility in these mutant mice.

In this study, we investigated the role of fidgetin in meiotic oocytes and female reproduction of mice. Our results demonstrated that fidgetin was predominant in the ovary and important for preventing polyspermy. However, the fidgetin knockout (KO) in mice did not impact female fertility, probably due to a compensation mechanism.

## Materials and methods

### Animal models

CRISPR/Cas9 technology was used to create the global fidgetin KO C57/B6 mice. Two pairs of sgRNA in exon 4 of *Fign* (*Supplementary Table 1*, available online), which contain four 20-base, gene-complementary oligos, were designed. Each oligo was inserted into pUC57-T7-gRNA. Linearized pUC57-T7-*Fign* gRNA were used as a template and purified it with a MEGAclear Kit (ThermoFisher Scientific, USA) to produce sgRNA with a MEGAshortscript Kit (ThermoFisher Scientific). We used an mMessage mMachine T7 kit (ThermoFisher Scientific) to produce the Cas9 mRNA, using linearized pST1374-N-NLS-flag-linker-Cas9 as a template.

We then utilized a poly A-tailing kit (ThermoFisher Scientific) and purified with a RNeasy Micro kit (Qiagen, Germany) to create mRNA tailed poly A, which increased mRNA stability. SgRNA and cas9 mRNA were sent to Animal Core Facility (ACF) in Nanjing Medical University (NJMU). Pronuclear microinjection, embryo transfer, and mouse parturition were then conducted. We detected genotypes of fidgetin KO mice using PCR and DNA sequencing. The forward primer was 5′-CAGCAGCCAGTCCAGTAGG-3′; the reverse primer was 5′-TCTCCGCTTCTCCTATCCAC-3′. The genotyping PCR program was as follows: 94 °C for 5 minutes, 32 cycles of melting at 94 °C for 30 seconds, annealing at 57.7 °C for 30 seconds, and extension at 72 °C for 60 seconds, with a final additional extension at 72 °C for 7 minutes.

For all fertility assays, we used 4 wild type (WT) and 4 fidgetin KO B6 female mice. WT mating male mice need to be rotated on a monthly basis between cages and according to a random allocation table (***Supplementary Table 2***, available online). Mating was initiated between fidgetin KO or WT female mice and WT male mice when they were aged two months.

Experimental animal procedures in this study were approved by NJMU's Animal Ethics Committee (AEC) (Approval No. IACUC-1903028). All mice were fed with standard specific pathogen-free conditions at the ACF. When the uterus or ovaries were removed, mice were anesthetized with CO_2_ and then euthanized by cervical dislocation.

WT and fidgetin KO oocytes were obtained from C57/B6 strain, and all other oocytes were collected from three-week-old WT CD1/ICR mice.

### Antibodies

Primary antibodies: anti-β-actin antibody (Cat# A5316-100), rabbit anti-katanin p60 AL2 antibody (N-15) (Cat# sc-84855), goat anti-katanin p60 A1 antibody (M-13) (Cat# sc-10929), mouse anti-katanin p60 AL1 antibody (A-10) (Cat# sc-373814), rabbit anti-fidgetin antibody (H-146) (Cat# sc-68343), goat anti-FIGNL1 antibody (C-12) (Cat# sc-138278), goat anti-FIGNL2 antibody (G-14) (Cat# sc-242820), mouse anti-β-tubulin antibody (Cat# sc-5274), and rabbit anti-ZP3 antibody (H-300) (Cat# sc-25802) were purchased from Santa Cruz (USA); mouse anti-spastin antibody (Cat# S7074) was purchased from Sigma (USA); mouse anti-5-methylcytosine antibody (Cat# ab73938) was purchased from Abcam (England). Another rabbit anti-fidgetin antibody was custom-made (antigen sequence: GLTPIAPSALTNNSA) and purified by Zoonbio Biotechnology (China).

Secondary antibodies: HRP-conjugated anti-rabbit IgG and anti-mouse IgG (Vazyme, China), Cy2-conjugated anti-mouse IgG (Cat# 715-225-150), Cy2-conjugated anti-rabbit IgG (Cat# 715-225-152), rhodamine-conjugated anti-mouse IgG (Cat# 715-025-150), all purchased from Jackson ImmunoResearch Laboratory (USA).

### Western blotting

Proteins were separated in 10% polyacrylamide gels and transferred onto a PVDF membrane using a highly efficient wet protein transfer system (Cat# L00686, GenScript, China). The PVDF membrane was blocked with 5% non-fat milk diluted with TBST (TBS containing 0.1% Tween-20) for 1 hour at room temperature and then incubated with primary antibody (diluted with 5% nonfat milk) overnight at 4 °C. The PVDF membrane was washed three times, incubated with HRP-conjugated secondary antibody (diluted with 5% nonfat milk) for 1 hour at room temperature, washed three times, and finally developed by an enhanced chemiluminescence (Biosharp, China) and detected by a Digital chemiluminescence imaging system (4500SF, Tanon, China).

### Semi-quantitative RT-PCR and quantitative real-time RT-PCR

For semi-quantitative reverse transcription (RT)-PCR, total RNA of mouse ovary was extracted by using Animal Total RNA Isolation Kit (Cat# RE-03014, Foregene, China). A total of 1 μg RNA was reversely transcribed to cDNA using HiFiScript gDNA Removal RT MasterMix (Cat# CW2020S, CWBIO, China). PCR was performed with the following primers: *Spast* forward, 5′-TTCTTTCTCGTCCCCGCTG-3′, reverse, 5′-TCCTCGTCGATGCGCAG; *Katna1* forward, 5′- TGCGCTAGCTTACATATGACC-3′, reverse, 5′-CTCTGACGACTGTGCTTGG; *Katnal1* forward, 5′- GGCGGAGATTTGTGAGAATGC-3′, reverse, 5′-TGGCCATACTGCAGGGTCTC; *Katnal2* forward, 5′- AAGAAGGCTACATGGATGCAG-3′, reverse, 5′-TTGTTCTTCCCTCCACTTCG; *Fign* forward, 5′- ACTCCGTGCAGCTACCTC-3′, reverse, 5′-GGTCTTGACCTGGTGAAGG; *Fignl1* forward, 5′- GGTGGACGAATGGCAGAAG-3′, reverse, 5′-GTCGAGATCCTGCCAAAGC; *Fignl2* forward, 5′- CAACCTCCTCAAGCGCTACG-3′, reverse, 5′-GCGTTGCCCGTGTACAGC; *Gapdh* forward, 5′-ACCACAGTCCATGCCATCAC-3′, reverse, 5′-CACCACCCTGTTGCTGTAGC-3′. PCR products were separated by 2% agarose gel.

For quantitative real-time RT-PCR, total RNA and cDNA were obtained as above. Gene expression was analyzed by using ChamQ SYBR qPCR Master Mix (Cat# Q711-02, Vazyme). The following primer pairs were used for quantitative real-time RT-PCR: *Spast* forward, 5′- CCAAGGACCGTTTACAACTTCT-3′, reverse, 5′-ATTGCGGCATGTCAGGTTAGT; *Katna1* forward, 5′-CAGTCAAAGATACACACCTCCG-3′, reverse, 5′-CTCAACAGGTACAGGCAAGGA; *Katnal1* forward, 5′- TCAAATCAGGCGTCCAAATC-3′, reverse, 5′-CCTTGTCATCTCTCCCTCTTGC; *Katnal2* forward, 5′- ATTACGGCGCTTTGAAGTTTG-3′, reverse, 5′-CCTCCACTTCGTGACGGTAAAT; *Fign* forward, 5′-CACACACCTCATTGACCTG-3′, reverse, 5′-GACCTCAGCACTGGCCAC; *Fignl1* forward, 5′- TTGGCAGGATCTCGACAGG-3′, reverse, 5′-GGTTCCAACAGAGACTCTTCAAA; *Fignl2* forward, 5′- CCCTAAACCAGTGGCCAGAG-3′, reverse, 5′-GCGGAAATGTCGTCGTGTG. Primers for *Gapdh* are same to that used for semi-quantitative RT-PCR.

### Immunofluorescence staining of oocytes

We used phosphate-buffered saline (PBS)/polyvinylpyrrolidone (PVP) for three quick washes. Fifty oocytes were permeated with 0.5% Triton X-100/PHEM for 5 minutes; this solution contains 60 mmol/L PIPES, 25 mmol/L Hepes, pH 6.9, 10 mmol/L EGTA, and 8 mmol/L MgSO_4_. After three quick washes at room temperature, the oocytes were incubated with 3.7% PFA for 20 minutes and then washed with PBS/PVP 3 times and for 10 minutes each. The oocytes were sealed with bovine serum albumin (BSA) (1% BSA and 100 mmol/L glycine in PBS) at room temperature for 1 hour. Primary antibody diluted with BSA was added to the oocytes and placed in a wet box for overnight incubation at 4 °C. Then, the oocytes were washed with PBST (0.05% Tween-20 in PBS) 3 times for 10 minutes each time.

The second fluorescent antibody, diluted with BSA, was added to the oocytes and incubated at room temperature for 45 minutes in the dark. A wash with PBST for 3 times for 10 minutes each time was then performed. The oocytes were incubated with 10 ng/μL DAPI (Cat# D9542, Sigma) diluted with PBST for 10 minutes. A quick wash with PBST was carried out 3 times; antifade was then added, and the tablet was sealed. Specimens were imaged on an Andor Revolution spinning disk confocal system (Andor Technology PLC, UK) using IQ2 (the software installed by the manufacturer to control all operations of the confocal). The system was mounted on an inverted TiE microscope (Nikon, Japan), using a 60× 1.4 NA objective lens, and images were captured with a cold CCD camera (Andor). Most images are displayed as a maximum intensity projection of the captured Z stack.

### Immunoprecipitation

The 30 μL protein- A/G beads (Cat# 36403ES03, Yeasen Co., China) were washed three times with 300 μL immunoprecipitation (IP) buffer (1 mmol/L EGTA, 20 mmol/L Tris-HCl, pH 8.0, 150 mmol/L NaCl, 10 mmol/L EDTA, 1 mmol/L phenylmethylsulfonyl fluoride, 0.05% Triton X-100, 0.05% Nonidet P-40) with 1:500 phosphatase inhibitor and 1:100 protease inhibitor, centrifuged at 200 *g* for 1 minute at 4 °C. For IgG-protein-A/G coupling, 5 μg rabbit anti-fidgetin antibody or control rabbit IgG were coupled to cleaned 30 μL protein-A/G beads in 250 μL IP buffer and rotated on a flip shaker for 4 hours at 4 °C. Next, the IgG-coupled protein A/G was washed three times (10 minutes each) and centrifuged at 200 *g* for 1 minute.

At the same time, for oocyte lysate precleaning, 600 oocytes were lysed in 250 μL IP buffer by ultrasound, then 30 μL cleaned protein-A/G beads were added to oocyte lysis solution and rotated on a flip shaker for 4 hours at 4 °C. Next the mixture was centrifuged at 200 *g* for 1 minute at 4 °C and the oocyte lysate was kept. Then the immunoprecipitation was performed by adding the IgG-coupled protein-A/G beads into the precleaned oocyte lysate and rotated on a flip shaker overnight at 4 °C. After that, the immunocomplex was washed three times in 250 µL IP buffer and boiled in protein sample buffer (Cat# BD0034-3, Bioworld Inc., USA) for 5 minutes, followed by SDS-PAGE.

### siRNA-mediated fidgetin knockdown in oocytes

*Fign* DNA templates for small interfering RNA (siRNA) are shown in ***Supplementary Table 3*** (available online). The siRNAs were produced and purified using the T7 Ribomax Express RNAi System (Promega, USA) according to the manufacturer's instructions. The siRNAs were checked for their level of purification *via* agarose gel electrophoresis and then stored at −80 °C. The siRNAs were mixed with four different target sites at the same molar ratio and a final concentration of 5 µmol/L siRNA to form a ready-to-use mixture of siRNA.

We utilized an N-TER nanoparticle siRNA transfection system (Sigma) per the manufacturer's instructions. Throughout the siRNA treatment process, generally 36 to 44 hours, 2.5 mmol/L milrinone was added to prevent meiosis recovery. Then, we collected 100 oocytes for experimentation.

### *In vitro* fertilization

Epididymal sperm from 10–18 week-old male mice (B6-DBA2 F1) were collected and capacitated with 1 mL MEM+ (MEM supplemented with 0.23 mmol/L sodium pyruvate, 0.01 mmol/L EDTA tetrasodium, 3 mg/mL BSA) for 1 hour. Then, 10 μL of suspension containing 5–10 μL sperm (10^6^/mL) was added to 490 μL MEM+ medium, and 50 oocytes washed from fetal bovine serum (FBS, ThermoFisher) were added. After 5 hours, a pipette was used to wash away any remaining sperm on the oocyte surface. Four hours later, the oocytes were immunoassayed to determine the frequency of successful fertilization by identifying prokaryotic formation.

### Oocyte collection and *in vitro* culture

Fully grown germinal vesicle (GV) oocytes were collected in 3-week-old female WT and fidgetin KO mice, respectively. One hundred oocytes were released by puncturing the ovaries with a sterile syringe needle in MEM+ medium (0.01 mmol/L EDTA, 0.23 mmol/L sodium pyruvate, 0.2 mmol/L penicillin/streptomycin, and 3 mg/mL BSA in MEM). After washing away cumulus cells from the cumulus-oocyte complex, cumulus-free oocytes were added to 100 µL of MEM+ droplets containing 10% FBS incubate and then covered with mineral oil. These oocytes were then cultured in a 37 °C incubator with a humidified atmosphere of 5% CO_2_ and 5% O_2_.

### Cell culture

NIH3T3 cells are a product of ATCC (Cat# CRL-1658) sold by Procell Life Science & Technology Co., Ltd. (China). NIH3T3 cells were cultured in Dulbecco's minimum essential medium (DMEM, Cat# BC-M-005, Biochannel, China) supplemented with 10% FBS in a 37 °C incubator with a humidified atmosphere of 5% CO _2_ and 5% O_2_.

### Silver staining and characterization of fidgetin-interacting proteins

Immunocomplexed beads from the control IgG and fidgetin antibody panels were boiled in protein sample buffer, respectively, and 5 μg were loaded side by side on an SDS-PAGE gel. The gel was first fixed overnight in 40% ethanol and 10% acetic acid, then sensitized with fresh sensitization solution (0.314% Na_2_S_2_O_3_∙5H_2_O and 6.8% sodium acetate, 30% ethanol, 0.2% Na_2_S_2_O_3_) for 30 minutes at room temperature. The gel was washed 3 times for 5 minutes each and then stained with staining solution (0.25% AgNO_3_, 0.02% fresh 37% formaldehyde solution) for 20 minutes at room temperature and washed with water for 2.5 minutes. Gels were developed in developer solution (2.5% NaCO_3_, 0.02% fresh 37% formaldehyde solution) for approximately 5–10 minutes. Finally, the development reaction was terminated with 0.4% glycine. The experiment was repeated three times.

### mRNA production and microinjection

Fidgetin and EGFP full-length coding sequences were amplified and cloned into pBluescript II SK (+) *Bam*HI/*Eco*RV and *Eco*RV/*Xho*I restriction sites for fidgetin-EGFP fusion expression. The plasmid was linearized by *Psi*I digestion and purified as a DNA template for fidgetin-EGFP mRNA transcription. The initial fidgetin-EGFP mRNA was transcribed using the T3 mMessage mMachine Ultra Kit (Ambion, USA), followed by the Poly(A) Tailing Kit (Ambion) to extend the 3′ UTR and stabilize the mRNA.

Microinjection was performed using a micropipette Puller P-97 (Sutter Instruments, USA). The tip of the needle was bent 30 degrees using a Micro Forge (Narishige CO., Japan). MPP6-EGFP mRNA (500–1000 ng/µL) was loaded into the front of the tip by wire-assisted siphoning. The needle was then loaded onto the 3-D electromechanical arm of a micromanipulator (Narishige) mounted on a Ti-S inverted fluorescence microscope (Nikon) and connected to a nitrogen-driven programmable injector (Narishige). The injection time was approximately 10–20 milliseconds, and the injection volume was about 10–20 pL. The experiment was repeated three times.

### Animal/individual sample inclusion, experiment grouping, data collection, and data analysis

All selected oocytes were of standard quality (fully grown oocytes with typical diameter, and zona pellucida [ZP] closely connected with the oocyte membrane, *etc.*). All selected female mice had to have been in good health in terms of average weight, regular diet, everyday activity, etc. All poor quality and unhealthy oocytes, and mice were excluded.

A blind approach was adopted in all experiments. Data acquisition, analysis, and recording were performed by different researchers.

For fertility determination, all mating cages were marked with the only cage number without any marks indicating "WT" or "fidgetin KO". A researcher inspected all cages, recorded the newborn, and sent the data to another researcher daily, who entered experimental data into a Microsoft Excel file for fertility measurement.

For other experiments, control and treatment (fidgetin KO and fidgetin knockdown [KD]) samples were exactly marked. During image acquisition, follicle count, intensity quantification, and grouping information of all samples were covered with black tapes and relabelled. After processing and removing tapes, a researcher was able to discover the correlation of analysis data with sample information quickly and record it.

Each individual (oocyte, ovary, mouse) in a separate repetition or group was selected for random and blind assignment prior to experimental manipulation. For independently repeated data collection, each data point was randomly selected.

### Statistical analysis

All statistical graphs for Western blotting, semi-quantitative RT-PCR, or quantitative real-time RT-PCR were from three independent repeats unless otherwise stated. The amount of protein loaded into each lane was about 50 μg in 30 μL sample buffer. All experiments were repeated at least three times. Data are presented as mean±SEM. Comparisons between two groups were made using a Student's *t*-test. Differences among more than two groups were compared using one-way ANOVAs. *P*<0.05 was considered statistically significant. Statistical analyses were conducted with GraphPad Prism.

## Results

### Mouse fidgetin showed a unique ZP-dominant localization among all MTSPs within oocytes

**Figure 1:**
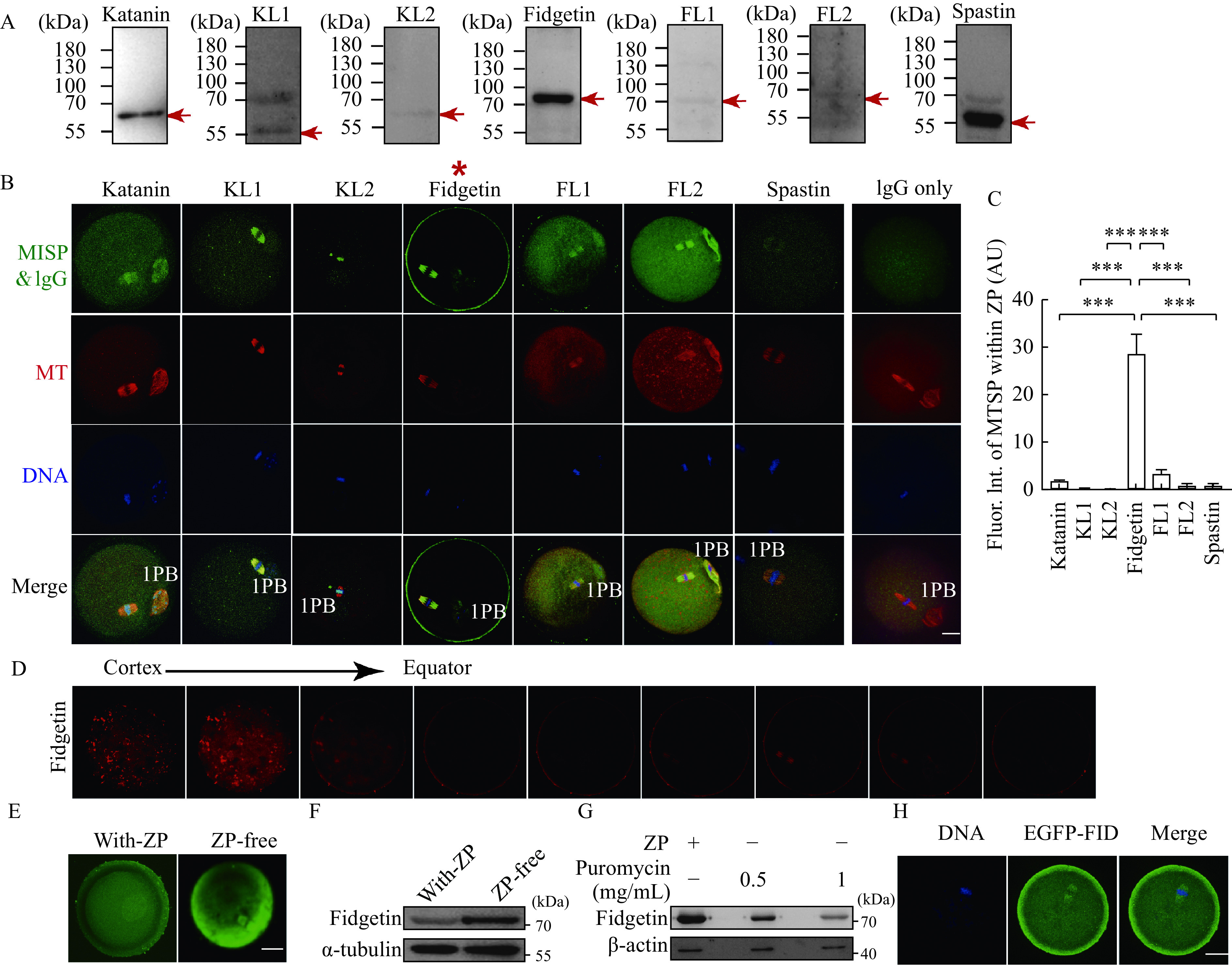
Mouse fidgetin has a unique ZP-dominant localization among all MTSPs within oocytes.

There are seven MTSPs in mice: katanin, KL1, KL2, fidgetin, fidgetin-like 1 (FL1), FL2, and spastin. We systematically examined the localization of these MTSPs within metaphase Ⅱ (MⅡ) mouse oocytes. Western blotting showed that all seven MTSP antibodies had good specificity (***Fig. 1A***). Immunofluorescence showed that they were fairly distinct from each other, although they shared some similarities (***Fig. 1B***). Interestingly, besides colocalization with spindle microtubules, only fidgetin had dominant enrichment within the ZP (***Fig. 1B***, red star, ***C***), as clearly shown by a consecutive Z-scan series of images from the ZP to the equator of the oocytes (***Fig. 1D***).

To further verify the ZP enrichment of fidgetin, we removed the ZP using an acidic (pH 2.5 adjusted by HCl) M2 medium (Sigma) and examined the fidgetin protein level. The results showed that a short time (5–10 seconds) ZP-removal treatment significantly increased fidgetin levels within oocytes, suggesting that fidgetin translation was rapidly upregulated in response to ZP removal (***Fig. 1E*** and ***F***). Furthermore, immunofluorescence demonstrated that a majority of increased fidgetin was focused beneath the cortex (***Fig. 1E***). Hence, we inhibited the protein translation 1 hour before ZP removal using puromycin and found that the short puromycin did not significantly affect β-actin translation, whereas the fidgetin level did decrease significantly (***Fig. 1G***). Finally, we *in vitro* transcribed and purified EGFP-Fidgetin mRNA and injected the mRNA into oocytes. We found that exogenous EGFP-fidgetin protein was translated from EGFP-Fidgetin mRNA, as detected by anti-EGFP antibody, further suggesting ZP enrichment of fidgetin (***Fig. 1H***).

**Figure 2:**
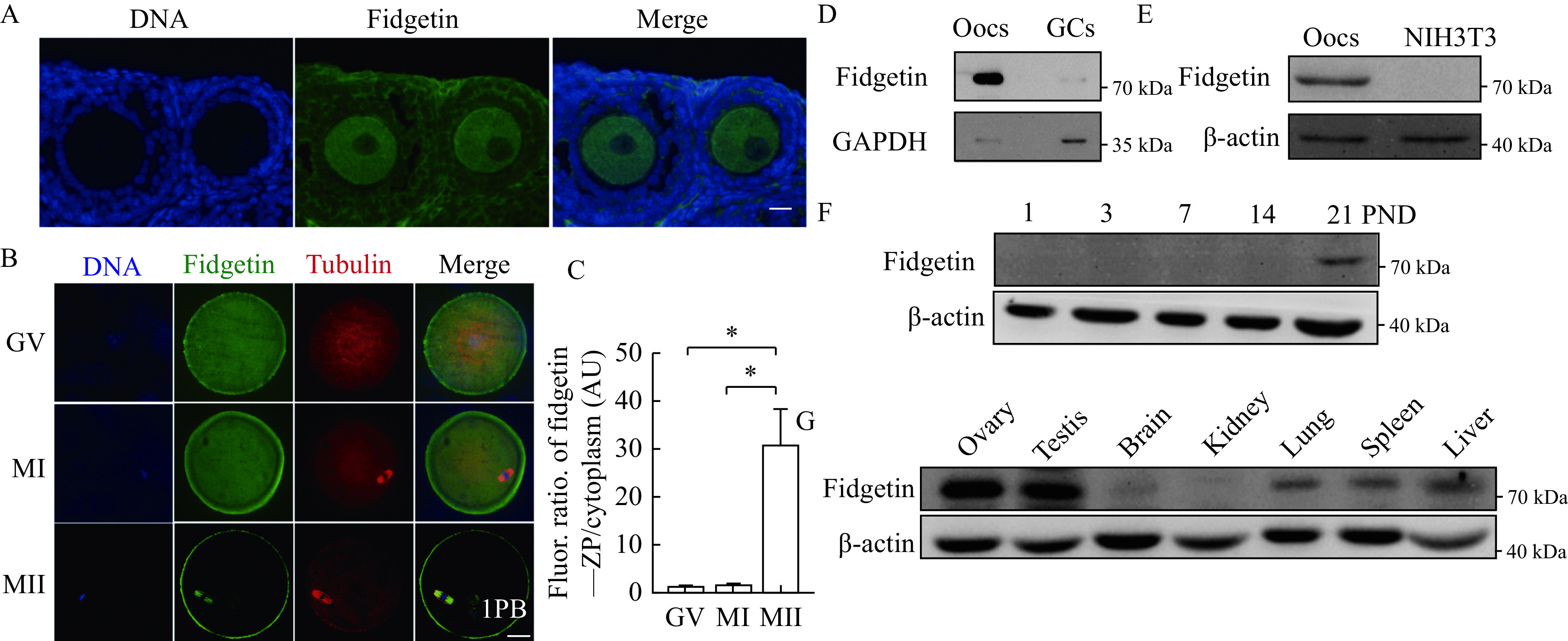
Mouse fidgetin is enriched within ovaries and oocytes.

We then examined whether fidgetin possessed the characteristics of the maternal protein. Ovarian immunofluorescence showed that fidgetin was more dominant within oocytes than within granulosa cells (GCs) (***Fig. 2A***). However, compared with *in vitro* maturation MII oocytes (***Fig. 1B***), the membrane enrichment within the GV oocytes of antral follicles was less prominent (***Fig. 2A***). We speculated that fidgetin's sub-cellular localization during meiosis was dynamic; therefore, we did parallel immunostaining for fidgetin at distinct meiotic stages. Quantification of the fluorescence ratio showed that fidgetin significantly increased the ZP enrichment from GV to MII, probably by re-localizing from the cytoplasm into ZP (***Fig. 2B*** and ***C***).

Next, Western blotting assays showed that fidgetin was much more predominant in oocytes than in GCs (***Fig. 2D***) or somatic cells (NIH3T3) (***Fig. 2E***), and the fidgetin level sharply increased within the ovary on post-natal day 21 (***Fig. 2F***), when the first wave of follicle recruitment and maturation occurs. Finally, fidgetin was more abundant within the ovary and testis than in other organs (***Fig. 2G***).

All the above results suggested that fidgetin might be important for female reproduction, although it is not a strictly defined maternal protein. In addition, its function might be closely correlated with its unique ZP enrichment.

### Fidgetin interacted with ZP proteins

**Figure 3:**
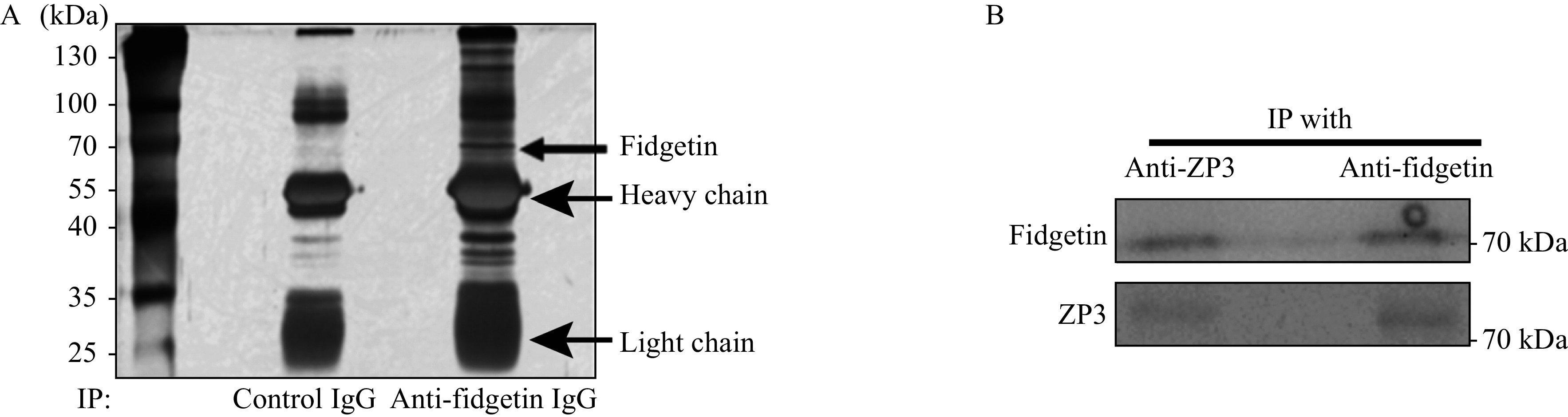
Fidgetin interacts with ZP proteins.

As fidgetin is enriched within ZP, we proposed that this might interact with other known ZP proteins. Therefore, we used a ZP3 antibody side by side with control IgG to perform IP and run SDS-PAGE. We then did silver staining on the gel and did find that there were many differential bands in the fidgetin IP lane in contrast to the control IP lane, suggesting that fidgetin interacts with many proteins (***Fig. 3A***). Next, gel regions beside antibody heavy chain and light chain were cut and sent for liquid chromatography-mass spectrometry (LC-MS). We identified all three ZP proteins as reliable fidgetin interactors (***Supplementary Table 4***, available online). Next, we were able to verify the interaction between fidgetin and ZP3 by co-immunoprecipitation (Co-IP) with anti-ZP3 and anti-fidgetin antibodies in oocyte lysate (***Fig. 3B***). This result indicated that fidgetin might be an intrinsic ZP protein essential for the primary function of ZP.

### Fidgetin knockdown increased polyspermy

**Figure 4:**
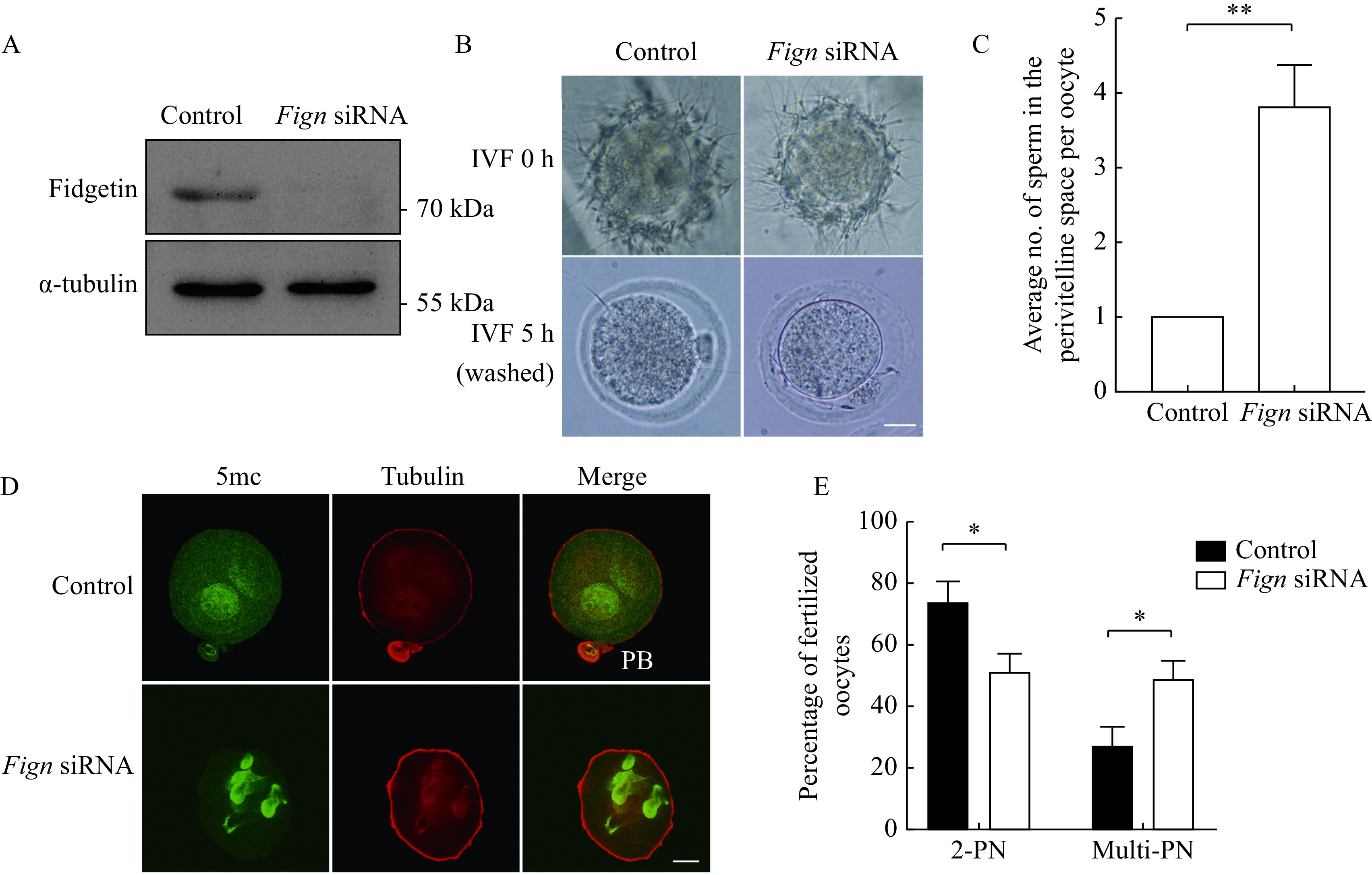
Fidgetin knockdown caused increased polyspermy.

The enrichment of fidgetin in ZP led us to hypothesize that fidgetin is essential for preventing polyspermy. To support this claim, we carried out fidgetin KD with siRNA against the 5′ UTR of *Fign*, which was confirmed by Western blotting (***Fig 4A***, ***Supplementary Table 3*** [available online]), followed by *in vitro* fertilization (IVF). The results showed that 5 hours after IVF, fidgetin KD significantly increased the number of sperm that penetrated the ZP into the perivitelline space or onto the oocyte membrane (***Fig. 4B*** and ***C***). Next, 5-methylcytosine (5mc, which labels the male pronucleus) immunofluorescence of IVF oocytes revealed that nine hours after IVF, fidgetin KD significantly decreased the number of normally fertilized oocytes with two pronuclei (2PN) but increased the number of abnormally fertilized oocytes with multi pronuclei (number of PN ≥3) (***Fig. 4D*** and ***E***). This result indicated that FIGN might function by preventing polyspermy.

### Fidgetin knockout did not affect female fertility

**Figure 5:**
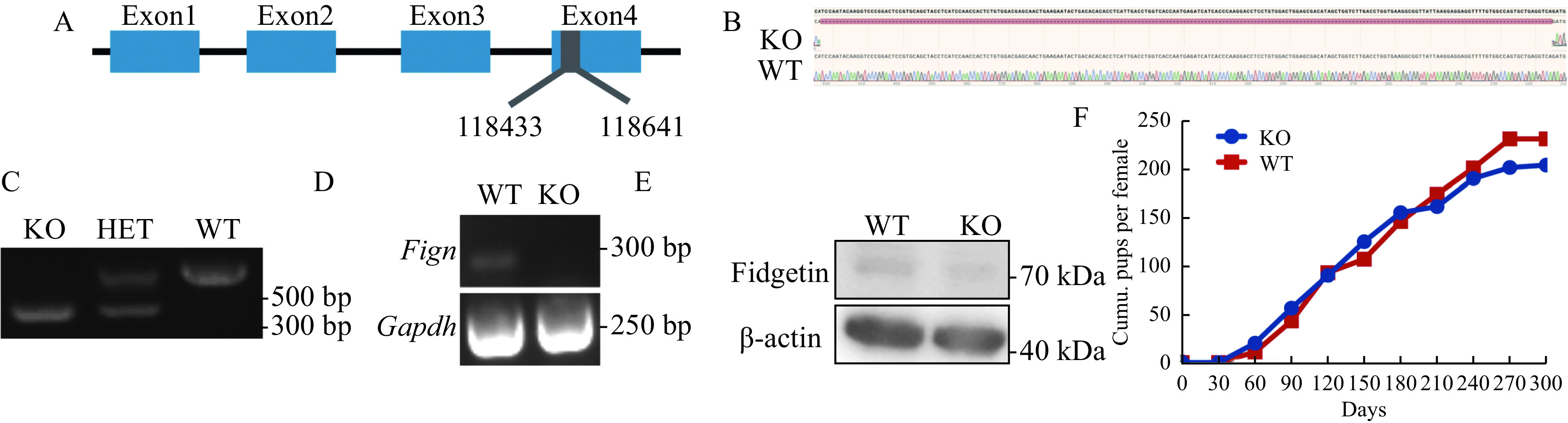
Fidgetin knockout did not affect female fertility.

To further investigate how fidgetin could prevent polyspermy, we use CRISPR/Cas9 to make global fidgetin KO mice by deleting 209 base pairs in exon 4, causing a frame shift (***Fig. 5A***), and Sanger sequencing and PCR were performed to verify the genotype (***Fig. 5B*** and ***C***). RT-PCR showed that *Fign* mRNA completely disappeared in the fidgetin KO ovary (***Fig. 5D***), and Western blotting revealed that fidgetin protein was also absent in fidgetin KO ovaries (***Fig. 5E***). However, fidgetin KO female mice were completely healthy (data not shown), and 9-month-long mating assays (fidgetin KO female mice mating with wild-type male mice) did not show any difference in the cumulative pups per female between KO and WT female mice (***Fig. 5F***). This result indicated that long-term global fidgetin deletion at the animal level and short-term fidgetin inhibition at the cell level impacts very distinctly.

### Fidgetin knockout specifically induced compensative upregulation of fidgetin-like 1

**Figure 6:**
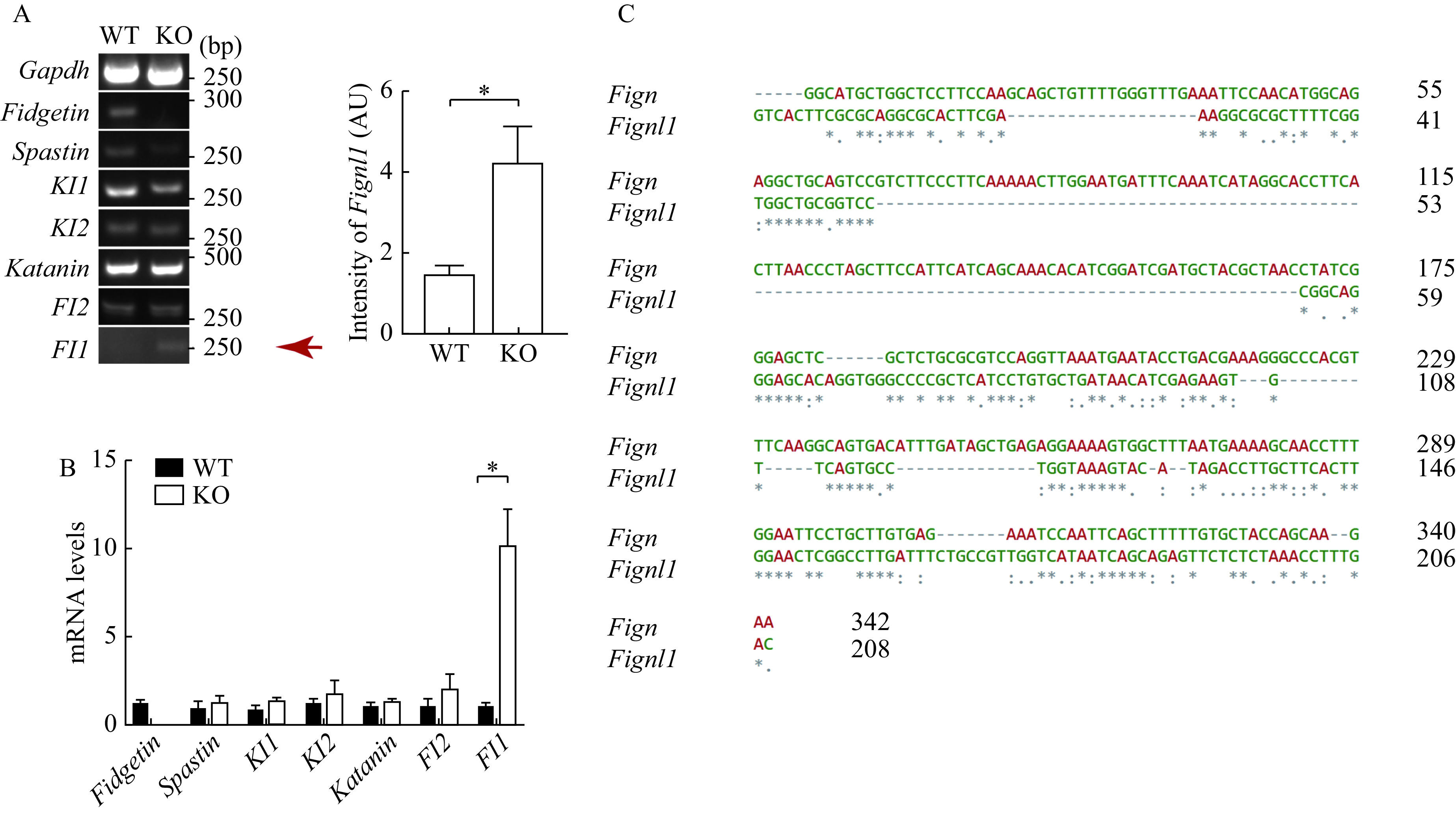
Fidgetin knockout specifically induced compensative upregulation of fidgetin-like 1.

The discrepancy between fidgetin KD and KO suggested that other members might compensate for fidgetin's function. Hence, we examined the mRNA levels of all MTSPs between WT and fidgetin KO mouse ovaries. RT-PCR and Q-PCR verified that only the *Fignl1* (which encodes FL1) mRNA level was significantly upregulated by fidgetin KO (***Fig. 6A*** and ***B***). Sequence alignment showed that 176–342 bp 5′ UTR of *Fign* mRNA shared relatively high similarity with 60–208 bp 5′ UTR of *Fignl1* mRNA (***Fig. 6C***). At the same time, *Fignl1* 5′ UTR mRNA shared very low similarity with other MTSPs (***Supplementary Fig. 1***, available online). This result indicated that distinctly from the short-term fidgetin inhibition at the cell level, the long-term global fidgetin deletion at the animal level conduced to the specific upregulation of FL1, which might compensate for fidgetin's function.

## Discussion

For the first time, we have shown that mouse MTSP, specifically fidgetin, has a unique ZP localization and might prevent polyspermy. We also demonstrated that fidgetin KO mice are physically and reproductively healthy, probably due to the compensatory action of FL1. The novelties can be generalized as follows:Firstly, for the first time, we did systematic immunostaining of all MTSPs in mouse oocytes and found that only fidgetin highly localized to ZP. As far as we know, no other studies compared the subcellular localization of all MTSPs in any type of cells. Secondly, we provided convincing evidences (fidgetin Western blotting after ZP removal and translation inhibition, localization of *in vitro* transcribed *Fign* mRNA within oocyte ZP, mass-spec identification of ZP1&ZP2&ZP3 as probable ZP-interacting proteins, and co-IP verification of the interaction between fidgetin and ZP3) showing that fidgetin might be a novel inherent ZP protein or closely associated with ZP. Thirdly, through multiple evidences (comparative fidgetin Western blotting of oocytes*vs.* granular cells, oocytes *vs.* somatic cells, ovaries of different PNDs, and different tissues; and comparative fidgetin immunostaining at different meiotic stages), we showed that fidgetin might be a novel maternal protein. Fourthly, we found that fidgetin knockdown caused polyspermy and further support that fidgetin might be important in the function of ZP. Fifthly, we found that, distinct from the in-vitro results, fidgetin KO didn't cause obvious effects on female fertility. Finally, we systematically examined the transcription of all MTSPs and found that only *Fignl1* mRNA level was significantly upregulated, and the 5′ UTR regions of *Fign* and *Fignl1* share high similarity, supporting that *Fignl1* might compensate for *Fign* in fidgetin KO mice. All these results provided solid foundation for further investigation of fidgetin's function in female reproduction.

Previous researchers have established that ZP comprises three ZP proteins (ZP1, ZP2, and ZP3). However, it could be that some ZP proteins, in relatively low abundance, were not successfully detected due to the limitations of mass spectrometry. In this study, we have preliminarily shown that fidgetin interacted with all three ZP proteins. Further strict characterization of ZP proteins will more clearly determine the inherent components of ZP. However, there are plenty of oocyte microvilli throughout the ZP, which will interfere with the characterization of ZP proteins. Another possibility is that, although fidgetin participates in the organization and function of ZP proteins, it is just associated with microvilli rather than being a fixed component of the ZP.

Fidgetin mutations are caused cell cycle delay in retinal cells^[10]^. Therefore, presumably fidgetin KO will affect oocyte meiosis and, ultimately, female fertility. However, in this study, we found that fidgetin KO did not influence female fertility in any observable way. Currently, we do not have a good explanation for the discrepancy between our results and previous reports. However, we propose that different gene knockout strategies might be the major cause. We saw that only *Fignl1* mRNA was significantly upregulated in fidgetin KO ovaries, suggesting that FL1 could compensate for the function of fidgetin.

FL1 had been shown to be a strong candidate for the impaired male meiosis and reduced testis weight in male mice^[12]^. FL1 has also been shown to be important for microtubule plus end dynamics^[13]^ and in homologous recombinations^[14–15]^. Interestingly, our findings suggest that fidgetin IP and LC-MS identify FL1 as the only interactor of fidgetin. The phenomena of deletion or mutation of one family member inducing the compensative upregulation of another family member have ever been reported. For example, human-induced pluripotent stem cells (hiPSCs) from fibroblasts of two patients carrying *SPG4* (which encodes the MTSP spastin) mutation had similar efficacy to control hiPSCs in differentiating into neurons and glia. Upregulation of another MTSP, p60 katanin, may partially compensate for the function (such as the regulation of microtubule dynamics) of fidgetin in *SPG4* mutant neurons^[16]^.

To date, researchers have reported the roles of MTSPs are all microtubule-associated^[17]^, but probably MTSPs can function on cytoskeleton and sub-cellular structure beyond microtubules. Our primary hypothesis is that in order for the sperm to penetrate ZP, ZP need reorganize into a tubular structure, while fidgetin will get activated to destabilize this structure and prevent the entry of the second sperm. However, this assumption needs extensive further investigation.

In summary, we have shown that fidgetin has a unique ZP localization among MTSPs and might act to prevent polyspermy. However, in fidgetin KO ovaries*,* the function of fidgetin might be successfully replenished by FL1. Further investigations are required to explore the mechanisms involved.

## References

[1] (2008). Structural basis of microtubule severing by the hereditary spastic paraplegia protein spastin. Nature.

[2] (2015). Microtubule severing by katanin p60 AAA+ ATPase requires the C-terminal acidic tails of both α- and β-tubulins and basic amino acid residues in the AAA+ ring pore. J Biol Chem.

[3] (2012). Microtubule-severing enzymes at the cutting edge. J Cell Sci.

[4] (2010). Microtubule-severing enzymes. Curr Opin Cell Biol.

[5] (2016). Microtubules and growth cones: motors drive the turn. Trends Neurosci.

[6] (2015). Microtubule nucleating and severing enzymes for modifying microtubule array organization and cell morphogenesis in response to environmental cues. New Phytol.

[7] (2002). Spastin, the protein mutated in autosomal dominant hereditary spastic paraplegia, is involved in microtubule dynamics. Hum Mol Genet.

[8] (2012). KATNAL1 regulation of sertoli cell microtubule dynamics is essential for spermiogenesis and male fertility. PLoS Genet.

[9] (2017). Katanin-like 2 (KATNAL2) functions in multiple aspects of haploid male germ cell development in the mouse. PLoS Genet.

[10] (2000). The mouse fidgetin gene defines a new role for AAA family proteins in mammalian development. Nat Genet.

[11] (2005). Functional characterization of fidgetin, an AAA-family protein mutated in fidget mice. Exp Cell Res.

[12] (2011). Fidgetin-like1 is a strong candidate for a dynamic impairment of male meiosis leading to reduced testis weight in mice. PLoS One.

[13] (2018). Motor axon navigation relies on Fidgetin-like 1-driven microtubule plus end dynamics. J Cell Biol.

[14] (2019). Antagonism between BRCA2 and FIGL1 regulates homologous recombination. Nucl Acids Res.

[15] (2013). FIGNL1-containing protein complex is required for efficient homologous recombination repair. Proc Natl Acad Sci U S A.

[16] (2014). Gene dosage-dependent rescue of HSP neurite defects in SPG4 patients' neurons. Hum Mol Genet.

[17] (2021). Cutting, amplifying, and aligning microtubules with severing enzymes. Trends Cell Biol.

